# Supplementary material for: Effect of a brief psychological intervention for common mental disorders on HIV viral suppression: A non-randomised controlled study of the Friendship Bench in Zimbabwe
Source: PLOS Glob Public Health. 2024 Jan 18;4(1):e0001492. doi: 10.1371/journal.pgph.0001492 (PMC10796049; doi:10.1371/journal.pgph.0001492)
Supplement: S1 Table — (DOCX) [file pgph.0001492.s001.docx]

**S1 Table: Associations between baseline characteristics and treatment group (exposure) or viral non-suppression at endline (outcome) presented as ORs with corresponding p-values from individual-level logistic regression analyses.**

|  | Association with treatment group | | | |  | Association with viral non-suppression at endline | | | |
| --- | --- | --- | --- | --- | --- | --- | --- | --- | --- |
|  | **Crude OR**  **(95% CI); N=579** | **p** | **Adjusted OR (95% CI); N=572** | **p** |  | **Crude OR (95% CI); N=579** | **p** | **Adjusted OR (95% CI) N=574** | **p** |
| SOCIODEMOGRAPHIC CHARACTERISTICS |  |  |  |  |  |  |  |  |  |
| Gender |  |  |  |  |  |  |  |  |  |
| Male | 1 | <0.001 | 1 | <0.001 |  | 1 | 0.81 | NA |  |
| Female | 2.09 (1.50, 2.91) |  | 2.06 (1.41, 3.01) |  |  | 1.13 (0.43, 2.99) |  |  |  |
| Marital status |  |  |  |  |  |  |  |  |  |
| Married | 1 | <0.001 | 1 | <0.001 |  | 1 | 0.16 | NA |  |
| Single | 1.03 (0.77, 1.36) |  | 0.85 (0.61, 1.17) |  |  | 1.55 (0.99, 2.44) |  |  |  |
| Widowed | 2.24 (1.59, 3.15) |  | 1.98 (1.44, 2.71) |  |  | 0.65 (0.38, 1.11) |  |  |  |
| Age |  |  |  |  |  |  |  |  |  |
| 18-29 | **1** | **<0.001 (0.79 trend)** | **1** | **0.006** |  | **1** | **0.01 (0.046 trend)** | **1** | **<0.001** |
| 30-39 | **0.82 (0.44, 1.53)** |  | **0.93 (0.41, 2.10)** |  |  | **0.83 (0.37, 1.85)** |  | **0.34 (0.12, 0.97)** |  |
| 40-49 | **0.73 (0.28, 1.92)** |  | **0.73 (0.22, 2.41)** |  |  | **0.38 (0.17, 0.85)** |  | **0.16 (0.05, 0.54)** |  |
| 50-59 | **1.13 (0.41, 3.16)** |  | **1.16 (0.35, 3.88)** |  |  | **0.18 (0.05, 0.62)** |  | **0.09 (0.03, 0.25)** |  |
| 60-72 | **1.22 (0.41, 3.59)** |  | **1.29 (0.33, 5.13)** |  |  | **1.01 (0.28, 3.60)** |  | **0.24 (0.03, 2.02)** |  |
| Highest level education achieved |  |  |  |  |  |  |  |  |  |
| Incomplete primary | 1 | 0.007  (0.68 trend) | 1 | <0.001 |  | 1 | 0.25 (0.63 trend) | NA |  |
| Complete primary | 0.53 (0.31-0.90) |  | 0.61 (0.26, 1.44) |  |  | 0.53 (0.13, 2.10) |  |  |  |
| Incomplete secondary | 0.92 (0.47-1.81) |  | 1.37 (0.63, 2.96) |  |  | 0.95 (0.32, 2.81) |  |  |  |
| Complete secondary | 0.46 (0.08-2.56) |  | 0.61 (0.06, 6.51) |  |  | 2.70 (0.36, 20.28) |  |  |  |
| Tertiary | 0.70(0.14-3.54) |  | 1.63 (0.39, 6.85) |  |  | 0.48 (0.05, 4.68) |  |  |  |
| Income (N=2 missing) |  |  |  |  |  |  |  |  |  |
| Yes | 1 | <0.001 | 1 | <0.001 |  | 1 | 0.43 | NA |  |
| No | 2.44 (1.50, 3.97) |  | 2.79 (1.72, 4.49) |  |  | 1.51 (0.55, 4.18) |  |  |  |
| Living in a house (N=5 missing) |  |  |  |  |  |  |  |  |  |
| No | 1 | 0.68 | NA |  |  | 1 | 0.76 | NA |  |
| Yes | 0.89 (0.50, 1.57) |  |  |  |  | 0.88 (0.38, 2.01) |  |  |  |
| Overcrowding ^$^ |  |  |  |  |  |  |  |  |  |
| No | 1 | 0.86 | NA |  |  | 1 | 0.60 | NA |  |
| Yes | 0.96 (0.63, 1.47) |  |  |  |  | 0.84 (0.44, 1.59) |  |  |  |
| Drinking alcohol |  |  |  |  |  |  |  |  |  |
| No | 1 | 0.32 | NA |  |  | 1 | 0.51 | NA |  |
| Yes | 0.74 (0.40, 1.35) |  |  |  |  | 1.19 (0.72, 1.96) |  |  |  |
|  |  |  |  |  |  |  |  |  |  |
| HIV RELATED CHARACTERISTICS | | | | | | | | | |
| Baseline HIV viral load |  |  |  |  |  |  |  |  |  |
| <400 copies/mL | 1 |  | NA |  |  | 1 |  | 1 |  |
| >400 copies/mL | 1.13 (0.81, 1.58) | 0.47 |  |  |  | 23.00 (11.05, 47.88) | <0.001 | 24.83 (11.70, 52.70) | <0.001 |
| Years since ART initiation (N=5 missing) |  |  |  |  |  |  |  |  |  |
| 0 | 1 | <0.001 (0.65 trend) | 1 | <0.001 |  | 1 | 0.23 (0.045 trend) | 1 | <0.001 |
| 1 | 0.84 (0.46-1.55) |  | 0.69 (0.32, 1.51) |  |  | 1.11 (0.50, 2.47) |  | 1.00 (0.39, 2.56) |  |
| 2 | 2.15 (1.59-2.92) |  | 2.05 (1.24, 3.37) |  |  | 1.84 (0.38, 8.80) |  | 0.94 (0.28, 3.19) |  |
| 3 | 0.63 (0.38-1.06) |  | 0.54 (0.30, 0.96) |  |  | 3.11 (0.95, 10.22) |  | 2.40 (0.54, 10.53) |  |
| 4 | 1.07 (0.71-1.62) |  | 0.83 (0.53, 1.30) |  |  | 1.35 (0.63, 2.88) |  | 2.10 (1.06, 4.18) |  |
| 5-9 | 0.89 (0.65-1.23) |  | 0.65 (0.36, 1.16) |  |  | 2.23 (0.80, 6.20) |  | 2.92 (1.16, 7.32) |  |
| 10-23 | 1.48 (1.05-2.07) |  | 1.37 (0.71, 2.64) |  |  | 2.83 (0.50, 15.91) |  | 6.31 (1.51, 26.28) |  |
|  |  |  |  |  |  |  |  |  |  |
| MENTAL HEALTH CHARACTERISTICS | | | | | | | | | |
| Depression (PHQ-9 ≥ 11) |  |  |  |  |  |  |  |  |  |
| No | 1 | 0.87 | NA | NA |  | 1 |  | 1 |  |
| Yes | 1.03 (0.70, 1.52) |  |  |  |  | 1.86 (1.22, 2.83) | 0.004 | 1.88 (1.03, 3.45) | 0.041 |
| Anxiety (GAD-7 ≥ 10) |  |  |  |  |  |  |  |  |  |
| No | 1 | 0.35 |  |  |  | 1 |  | NA |  |
| Yes | 1.21 (0.81, 1.82) |  |  |  |  | 1.04 (0.63, 1.69) | 0.89 |  |  |
| Risk identified* |  |  |  |  |  |  |  |  |  |
| No red flags | 1 | 0.05 | NA | NA |  | 1 |  | NA |  |
| Red flags | 1.21 (1.00, 1.47) |  |  |  |  | 1.44 (0.82, 2.53) | 0.21 |  |  |
| PHQ-9 (Patient Health Questionnaire): 0 (no symptoms) to 27 (worst possible symptoms)  GAD-7 (Generalized Anxiety Disorder 7-item Scale): 0 (no symptoms) to 21 (worst possible symptoms)  ^$^ includes index person  * SSQ-14 score ≥11 and either suicidal ideation or hallucinations | | | | | | | | | |
