# Supplementary material for: Effect of a brief psychological intervention for common mental disorders on HIV viral suppression: A non-randomised controlled study of the Friendship Bench in Zimbabwe
Source: PLOS Glob Public Health. 2024 Jan 18;4(1):e0001492. doi: 10.1371/journal.pgph.0001492 (PMC10796049; doi:10.1371/journal.pgph.0001492)
Supplement: S2 Table — (DOCX) [file pgph.0001492.s002.docx]

**S2 Table:** **Associations between baseline characteristics and completeness of viral load (VL) data presented as ORs with corresponding p-values from individual-level logistic regression analyses.**

|  | | Complete VL data  (N = 579) | Incomplete VL data  (N = 121) | OR (95% CI) | p-value |
| --- | --- | --- | --- | --- | --- |
| Gender | |  |  |  |  |
| Male | | 104 (18.0%) | 25 (20.7%) | 1 |  |
| Female | | 475 (82.0%) | 96 (79.3%) | 1.19 (0.74-1.90) | 0.47 |
| Marital status | |  |  |  |  |
| Married | | 279 (48.2%) | 69 (57.0%) | 1 | 0.046 |
| Single | | 154 (26.6%) | 28 (23.1%) | 1.36 (0.88-2.11) |  |
| Widowed | | 146 (25.2%) | 24 (19.8%) | 1.50 (1.07-2.12) |  |
| Age | |  |  |  |  |
| 18-29 | | 72 (12.4%) | 17 (14.1%) | 1 | 0.47 (0.54 trend) |
| 30-39 | | 200 (34.5%) | 43 (35.5%) | 1.10 (0.63-1.93) |  |
| 40-49 | | 206 (35.6%) | 45 (37.2%) | 1.08 (0.57-2.07) |  |
| 50-59 | | 79 (13.6%) | 11 (9.1%) | 1.70 (0.67-4.31) |  |
| 60-72 | | 22 (3.8%) | 5 (4.1%) | 1.04 (0.26-4.17) |  |
| Highest level education achieved | |  |  |  |  |
| Incomplete primary | | 31 (5.4) | 6 (5.0) | 1 | 0.99 (0.86 trend) |
| Complete primary | | 96 (16.6) | 22 (18.2) | 0.84 (0.22-3.24) |  |
| Incomplete secondary | | 430 (74.3) | 89 (73.6) | 0.94 (0.27-3.29) |  |
| Complete secondary | | 7 (1.2) | 1 (0.8) | 1.35 (0.10-18.52) |  |
| Tertiary | | 15 (2.6) | 3 (2.5) | 0.97 (0.15-6.07) |  |
| Income | |  |  |  |  |
| Yes | | 514 (89.1) | 112 (92.6) | 1 |  |
| No | | 63 (10.9) | 9 (7.4) | 1.53 (0.95-2.46) | 0.083 |
| Living in a house | |  |  |  |  |
| No | | 95 (16.6) | 17 (14.2) | 1 |  |
| Yes | | 479 (83.5) | 103 (85.8) | 0.83 (0.46-1.52) | 0.55 |
| Overcrowding^$^ | |  |  |  |  |
| No | | 500 (86.4) | 106 (87.6) | 1 |  |
| Yes | | 79 (13.6) | 15 (12.4) | 1.12 (0.58-2.15) | 0.74 |
| Drinking alcohol | |  |  |  |  |
| No | | 503 (86.9%) | 111 (91.7%) | 1 |  |
| Yes | | 76 (13.1%) | 10 (8.3%) | 1.68 (1.06-2.66) | 0.027 |
|  | |  |  |  |  |
| HIV CHARACTERISTICS | |  |  |  |  |
| Year since ART initiation | |  |  |  |  |
| 0 | | 91 (15.9) | 26 (21.7) | 1 | <0.001 (0.22 trend) |
| 1 | | 69 (12.0) | 11 (9.2) | 1.79 (0.80-4.03) |  |
| 2 | | 61 (10.6) | 10 (8.3) | 1.74 (1.02-2.98) |  |
| 3 | | 50 (8.7) | 14 (11.7) | 1.02 (0.53-1.95) |  |
| 4 | | 46 (8.0) | 10 (8.3) | 1.31 (0.68-2.54) |  |
| 5-9 | | 221 (38.5) | 45 (37.5) | 1.40 (0.90-2.18) |  |
| 10-23 | | 36 (6.3) | 4 (3.3) | 2.57 (1.32-5.02) |  |
|  | |  |  |  |  |
| MENTAL HEALTH CHARACTERISTICS | |  |  |  |  |
| Depression (PHQ-9 ≥ 11) | |  |  |  |  |
| No | | 256 (44.2) | 61 (50.4) | 1 |  |
| Yes | | 323 (55.8) | 60 (49.6) | 1.28 (0.85-1.94) | 0.24 |
| Anxiety (GAD-7 ≥ 10) | |  |  |  |  |
| No | | 307 (53.0) | 69 (57.0) | 1 |  |
| Yes | | 272 (47.0) | 52 (43.0) | 1.17 (0.85-1.62) | 0.33 |
| Risk identified* | |  |  |  |  |
| No red flags | | 297 (51.3) | 70 (57.9) | 1 |  |
| Red flags | | 282 (48.7) | 51 (42.2) | 1.30 (1.10-1.55) | 0.003 |
|  | PHQ-9 (Patient Health Questionnaire): 0 (no symptoms) to 27 (worst possible symptoms)  GAD-7 (Generalized Anxiety Disorder 7-item Scale): 0 (no symptoms) to 21 (worst possible symptoms)  ^$^ includes index person  * SSQ-14 score ≥11 and either suicidal ideation or hallucinations | | | | |
