# Supplementary material for: Effect of a brief psychological intervention for common mental disorders on HIV viral suppression: A non-randomised controlled study of the Friendship Bench in Zimbabwe
Source: PLOS Glob Public Health. 2024 Jan 18;4(1):e0001492. doi: 10.1371/journal.pgph.0001492 (PMC10796049; doi:10.1371/journal.pgph.0001492)
Supplement: Attachment — Submitted filename: rebuttal_ma2.docx [file pgph.0001492.s004.docx]

Dear editors,

**Re: Effect of a brief psychological intervention for common mental disorders on HIV viral suppression: a non-randomised controlled study of the Friendship Bench in Zimbabwe**

Thank you for your requested revisions of our paper. The points you have raised are addressed below in italics.

Journal Requirements:

1. Please include a complete copy of PLOS’ questionnaire on inclusivity in global research in your revised manuscript.

*A copy of the inclusivity questionnaire has been included.*

2. Please amend your detailed Financial Disclosure statement. This is published with the article. It must therefore be completed in full sentences and contain the exact wording you wish to be published.
   a. State what role the funders took in the study. If the funders had no role in your study, please state: “The funders had no role in study design, data collection and analysis, decision to publish, or preparation of the manuscript.”
      b. If any authors received a salary from any of your funders, please state which authors and which funders.

*The financial disclosure statement is as follows:*

*“This study was funded through a grant from the National Institute for Mental Health (NIMH), and The U.S. President's Emergency Plan for AIDS Relief (PEPFAR) awarded to DC. EM and LD received part-time salary from this PEPFAR/NIMH grant. VS and HAW are supported by the UK Medical Research Council (MRC) and the UK Department for International Development (DFID) under the MRC/DFID Concordat agreement which is also part of the EDCTP2 programme supported by the European Union (MR/R010161/1). The funders had no role in study design, data collection and analysis, decision to publish, or preparation of the manuscript”*

3. We ask that a manuscript source file is provided at Revision. Please upload your manuscript file as a .doc, .docx, .rtf or .tex.

*A revised .docx document file has been uploaded.*

4. Please provide separate figure files in .tif or .eps format. For more information about figure files please see our guidelines:  LINK

<https://journals.plos.org/globalpublichealth/s/figures> 
[https://journals.plos.org/globalpublichealth/s/figures#loc-file-requirements](https://protect-eu.mimecast.com/s/fiYLCnO14crGXzEHZ5pXT?domain=eur03.safelinks.protection.outlook.com)

*The figures are provided in .tif format.*

5. We noticed that you used “data not shown”/"unpublished data" in the manuscript. We do not allow these references, as the PLOS data access policy requires that all data be either published with the manuscript or made available in a publicly accessible database. Please amend the supplementary material to include the referenced data or remove the references.

*This reference has been removed.*

6. In the online submission form, you indicated that your data will be submitted to a repository upon acceptance.  We strongly recommend all authors deposit their data before acceptance, as the process can be lengthy and hold up publication timelines. Please note that, though access restrictions are acceptable now, your entire data will need to be made freely accessible if your manuscript is accepted for publication. This policy applies to all data except where public deposition would breach compliance with the protocol approved by your research ethics board. If you are unable to adhere to our open data policy, please kindly revise your statement to explain your reasoning and we will seek the editor's input on an exemption. Please be assured that, once you have provided your new statement, the assessment of your exemption will not hold up the peer review process.

*The dataset has been deposited in the LSHTM DataCompass repository and is currently under review.*

Additional Editor Comments (if provided):

- Nicely penned

*Thank you*

- Background may be written with more focus on rationale of doing the study

*The introduction section describes the evidence gap (whether treatment of depression alone without additional adherence support improves viral suppression) and the aim of this study, which evaluated the effect of a mental health intervention without additional adherence support on HIV viral suppression.*

- Impact of covid on immunosupression in mental health may be added in discussion

*This study was completed before the beginning of the COVID-19 pandemic so it did not affect the results.*

Yours sincerely,

Dr Vicky Simms and Prof Melanie Abas (on behalf of all authors)
