# Supplementary material for: Effect of a brief psychological intervention for common mental disorders on HIV viral suppression: A non-randomised controlled study of the Friendship Bench in Zimbabwe
Source: PLOS Glob Public Health. 2024 Jan 18;4(1):e0001492. doi: 10.1371/journal.pgph.0001492 (PMC10796049; doi:10.1371/journal.pgph.0001492)
Supplement: Attachment — Submitted filename: PGPH-D-22-02032_reviewer.docx [file pgph.0001492.s005.docx]

PLOS Global Public Health

# Effect of a brief psychological intervention for common mental disorders on HIV viral suppression: a non-randomised controlled study of the Friendship Bench in Zimbabwe

--Manuscript Draft--

| **Manuscript Number:** | PGPH-D-22-02032 |
| --- | --- |
| **Article Type:** | Research Article |
| **Full Title:** | Effect of a brief psychological intervention for common mental disorders on HIV viral suppression: a non-randomised controlled study of the Friendship Bench in Zimbabwe |
| **Short Title:** | Friendship Bench and HIV viral suppression |
| **Corresponding Author:** | Melanie Amna Abas, M.D. King's College London London, UNITED KINGDOM |
| **Order of Authors:** | Victoria Simms |
|  | Melanie Amna Abas |
|  | Monika Müller |
|  | Epiphania Munetsi |
|  | Lloyd Dzapasi |
|  | Helen A Weiss |
|  | Dixon Chibanda |
| **Keywords:** | HIV, depression, mental health, Adherence, Africa, Zimbabwe, task-shifting, cohort |
| **Abstract:** | Background: For people living with co-morbid HIV and common mental disorders (CMD), it is not known whether a brief psychological intervention for CMD can improve HIV viral suppression.  Methods: We conducted a prospective cohort study in eight primary care clinics in Harare, Zimbabwe, enrolling adults with co-morbid HIV and CMD. Six clinics provided the Friendship Bench (FB), a brief psychological intervention for CMD based on problem-solving therapy, delivered by lay counsellors. Two clinics provided enhanced usual care (EUC). The primary outcome was viral non-suppression after six months (viral load ≥400 copies/mL). Data were analysed using a difference-in-difference approach with linear regression of cluster-level proportions, adjusted for baseline viral non-suppression (aDiD). The secondary outcome was presence of CMD measured by the Shona Symptom Questionnaire.  Results: In FB clinics, 407/500 (81.4%) participants had viral load results at baseline and endline: 58 (14.3%) had viral non-suppression at baseline and 41 (10.1%) at endline . In EUC clinics, 172/200 (86.0%) had viral load results at baseline and endline: 22 (12.8%) were non-suppressed at baseline and 26 (15.1%) at endline (aDiD= -7.3%; 95%CI 14.7% to -0.01%; p=0.05). Of the 499 participants virally suppressed at baseline, the FB group had lower prevalence of non-suppression at endline compared to the EUC group (2.9% vs 9.3%; p=0.002). There was no evidence of a difference in endline viral non-suppression by group among the 80 participants with non- suppression at baseline (53.5% vs 54.6%; p=0.93). The FB group were less likely to screen positive for CMD at endline than the EUC group (aDiD= -21.6%; 95%CI -36.5% to -6.7%; p=0.008).  Conclusion: People living with co-morbid HIV and CMD may benefit from receiving a low-cost mental health intervention to enhance viral suppression, especially if they are already virally suppressed. Research is needed to understand if additional adherence counselling could further improve viral suppression. |
| **Additional Information:** |  |
| **Question** | **Response** |
| **Financial Disclosure** | This study was funded with support from the National Institute for Mental Health (NIMH), and the U.S. President's Emergency Plan for AIDS (PEPFAR). |

| Enter a financial disclosure statement that describes the sources of funding for the work included in this submission and the role the funder(s) played. This includes grants and any commercial funding of the work or authors.  This statement will be typeset if the manuscript is accepted for publication.  *Please review the* [*submission guidelines*](http://journals.plos.org/globalpublichealth/s/submission-guidelines#loc-financial-disclosure-statement) *and the instructions link below for detailed requirements and guidance.* |  |
| --- | --- |
| **Competing Interests**  On behalf of all authors, disclose any competing interests that could be perceived to bias this work.  This statement will be typeset if the manuscript is accepted for publication.  *Please review the instructions link below and PLOS Global Public Health’s* [*competing interests*](https://journals.plos.org/globalpublichealth/s/competing-interests) *policy to determine what information must be disclosed at submission.* | No potential competing interest was reported by the authors. |
| **Data Availability**  Before publication, Authors are required to make fully available and without restriction all data underlying their findings. Please see our [PLOS Data](https://journals.plos.org/globalpublichealth/s/data-availability) [Policy](https://journals.plos.org/globalpublichealth/s/data-availability) page for detailed information on this policy.  A **Data Availability Statement**, detailing where the data can be accessed, is required at first submission. Insert your Data Availability Statement in the box below. | The dataset, codebook and CRF have been submitted for review to the LSHTM DataCompass curated repository and we are awaiting confirmation of acceptance and a DOI. |

| Please see the [data reporting](https://journals.plos.org/globalpublichealth/s/submission-guidelines#loc-data-reporting) section of our submission guidelines for instructions on what you need to include in your Data Availability Statement.  This statement will be typeset if the manuscript is accepted for publication.  *PLOS allows rare exemptions to address legal and ethical concerns. If you have legal or ethical restrictions, please use the box below to detail these in full sentences for the Journal team to consider.* |  |
| --- | --- |

Manuscript	[Click here to access/download;Manuscript;renamed_89536.pdf](https://www.editorialmanager.com/pgph/download.aspx?id=123742&guid=bf128033-515e-4eb1-a3ff-e8cc2b75cbb8&scheme=1)

# 1 Effect of a brief psychological intervention for common mental

2 **disorders on HIV viral suppression: a non-randomised controlled study**

# 3 of the Friendship Bench in Zimbabwe

4

5 Victoria Simms*^1^ ¶, Melanie A Abas^2^*¶, Monika Müller^2,3,^, , Epiphania Munetsi^4^, Lloyd Dzapasi^4^,
6 Helen A Weiss^1^, Dixon Chibanda^4,5,6^

7

8 ^1^ MRC International Statistics and Epidemiology Group, London School of Hygiene & Tropical
9 Medicine, London, UK
10 ^2^ Centre for Global Mental Health, Institute of Psychiatry, Psychology and Neuroscience, King’s
11 College London, London, UK
12 ^3^ University Hospital of Psychiatry Bern, Bern, Switzerland
13 ^4^ Friendship Bench, Harare, Zimbabwe
14 ^5^ Department of Psychiatry, University of Zimbabwe, Harare, Zimbabwe
15 ^6^ Centre For Global Mental Health, London School of Hygiene and Tropical Medicine, London, UK

16

17	¶ these authors contributed equally to this work

18

19 Joint corresponding authors: Victoria Simms, Melanie A Abas
20 [victoria.simms@lshtm.ac.uk](mailto:victoria.simms@lshtm.ac.uk) (VS), [melanie.abas@kcl.ac.uk](mailto:melanie.abas@kcl.ac.uk) (MA)

21

22	Short running title: Friendship Bench and HIV viral suppression

23

24

## 25 Abstract

26 **Background**: For people living with co-morbid HIV and common mental disorders (CMD), it is not
27 known whether a brief psychological intervention for CMD can improve HIV viral suppression.
28 **Methods:** We conducted a prospective cohort study in eight primary care clinics in Harare,
29 Zimbabwe, enrolling adults with co-morbid HIV and CMD. Six clinics provided the Friendship Bench
30 (FB), a brief psychological intervention for CMD based on problem-solving therapy, delivered by lay
31 counsellors. Two clinics provided enhanced usual care (EUC). The primary outcome was viral non-
32 suppression after six months (viral load ≥400 copies/mL). Data were analysed using a difference-in-
33 difference approach with linear regression of cluster-level proportions, adjusted for baseline viral
34 non-suppression (aDiD). The secondary outcome was presence of CMD measured by the Shona
35 Symptom Questionnaire.
36 **Results:** In FB clinics, 407/500 (81.4%) participants had viral load results at baseline and endline:
37 58 (14.3%) had viral non-suppression at baseline and 41 (10.1%) at endline . In EUC clinics, 172/200
38 (86.0%) had viral load results at baseline and endline: 22 (12.8%) were non-suppressed at baseline

39	and 26 (15.1%) at endline (aDiD= -7.3%; 95%CI 14.7% to -0.01%; p=0.05). Of the 499 participants

40 virally suppressed at baseline, the FB group had lower prevalence of non-suppression at endline
41 compared to the EUC group (2.9% vs 9.3%; p=0.002). There was no evidence of a difference in
42 endline viral non-suppression by group among the 80 participants with non-suppression at baseline
43 (53.5% vs 54.6%; p=0.93). The FB group were less likely to screen positive for CMD at endline than

44	the EUC group (aDiD= -21.6%; 95%CI -36.5% to -6.7%; p=0.008).

45 **Conclusion:** People living with co-morbid HIV and CMD may benefit from receiving a low-cost
46 mental health intervention to enhance viral suppression, especially if they are already virally
47 suppressed. Research is needed to understand if additional adherence counselling could further
48 improve viral suppression.

49

## 50 Introduction

51 Sustained viral suppression is a key goal of care for people living with HIV (PLWH) because it halts
52 progression to AIDS, enables near-normal life expectancy and prevents transmission to sexual
53 partners [1]. Antiretroviral therapy (ART) taken as prescribed is the key driver of viral suppression
54 [2], with a recent systematic review finding that a diverse range of interventions are helpful in
55 improving ART adherence[3]. Common mental disorders (CMD), including depression and anxiety
56 increase risk of poor adherence to ART globally [4, 5], including in Zimbabwe [6], contributing to viral
57 non-suppression and mortality [7, 8]. CMD are very frequent in PLWH living in countries in sub-
58 Saharan Africa [9, 10]. A recent global systematic review reported 28-62% of PLWH have mental
59 health symptoms, especially depression, anxiety and suicidal ideation [11]. The WHO and UNAIDS
60 have recently emphasised the importance of addressing mental health in PLWH [12].

61

62 A recent systematic review of randomised controlled trials (RCT) found that task-shifted
63 psychological therapies are effective for mental disorders in PLWH in low- and middle-income
64 countries (LMICs) [13]. This review did not identify any RCTs from LMICs evaluating the
65 effectiveness of CMD treatment on ART adherence and/or viral suppression in PLWH [14]. A widely-
66 cited systematic review and meta-analysis, based almost exclusively on studies from high-income
67 countries, suggested that treatment of depression improves ART adherence (standardized relative
68 risk 1.35, 95% CI 1.13-1.60) [15]. However, many studies included in this review were prone to recall
69 bias or described interventions which included adherence counselling as well as depression
70 treatment. There remains an evidence gap on whether treatment of depression alone, without
71 additional adherence support, improves viral suppression.

72

73 The Friendship Bench (FB) is a brief psychological intervention for depression and other CMD based
74 on problem-solving therapy and simple behavioural activation [16]. The FB was developed in
75 Zimbabwe and is delivered by trained lay counsellors in primary care;[17]. A cluster-randomised
76 controlled trial showed that FB was effective at improving symptoms of CMD and of depression in
77 adults with and without HIV who accessed primary care [18].

78

79 The aim of this comparative cohort study was to evaluate the effect of FB on HIV viral suppression
80 and mental health outcomes among PLWH with co-morbid CMD in Harare, Zimbabwe. Our
81 hypothesis was that receiving the Friendship Bench, compared to usual care, is associated with viral
82 suppression at 6 months follow-up.

83

## 84 Materials and Methods

### 85 Study design and setting

86 The study was a multi-centre prospective cohort study of PLWH with co-morbid CMD, who attended
87 one of eight primary care clinics in high- or middle-density suburbs of Harare. When the study began
88 (August 2017), scale-up of the FB was taking place in primary care clinics in Harare. Six FB clinics
89 were purposively selected for their large patient volume (>300/day). Of the primary care clinics that
90 did not yet offer FB, the two largest in terms of patient volume were selected as enhanced usual
91 care (EUC) clinics. All clinics provided HIV care and other services including acute primary care,
92 chronic disease outpatients, family planning, and maternity at theprimary care level. None routinely
93 provided specialist mental health care. They were typically staffed by up to 50 health care
94 professionals with an average of 20 nurses and two physicians.

### 95 Recruitment

96 We used systematic sampling to select every third patient at each clinic (based on their allocated
97 queue number) to screen for eligibility. PLWH aged 18 years or above were eligible if they had been
98 on ART for at least three months, attended HIV care at the clinic in person, lived in the clinic
99 catchment area and had co-morbid CMD diagnosed using the Shona Symptom Questionnaire (SSQ-
100 14≥9) [19]. We excluded participants if they were receiving mental health care in a psychiatric unit,
101 presented with suicidal intent, psychotic symptoms, intoxication, or dementia. We defined
102 participants as ‘red flag’ cases if they had a SSQ-14≥11 or screened positive for either suicidal
103 ideation (“Did you sometimes feel like taking your own life”) or for psychotic symptoms ("Did you see
104 or hear things which others could not see or hear”). These participants were subsequently assessed
105 by a senior lay counselor with supervisor capacity and re-screened using the Patient Health
106 Questionnaire (PHQ-9) for depression. If participants were deemed by the senior counselor and the
107 study psychologist to have suicidal intent, or if psychotic symptoms were confirmed, they were not
108 eligible for the study and were referred for specialist care at a tertiary care facility. Those who were
109 eligible for the study were evaluated by the senior counsellor and referred back to the FB.
111 Eligible participants were asked for written informed consent to participate in the study which
112 included access to their routine medical records. Participants received US $3 for each study related
113 visit to cover transport costs. Ethical approval was granted by the Medical Research Council of
114 Zimbabwe (MRCZ A/2130) and the ethics committees of the London School of Hygiene & Tropical
115 Medicine (Ref 11759). The study observed the STROBE guideline for reporting on observational
116 studies [20].

### 117 Data collection at baseline and endline

118 Eight research assistants interviewed participants at baseline and at endline (six months post-
119 enrolment) using a tablet-based questionnaire to collect sociodemographic and mental health
120 outcomes. The questionnaire was developed based on similar studies previously conducted in this
121 setting [18, 19]. Participants who did not present for their endline visit were contacted by phone or
122 through significant others using contact data collected at baseline.
124 Socio-demographic characteristics included gender, age, marital status, education, income, type of
125 housing, number of persons sleeping in one bedroom including index person, and current alcohol
126 use. Overcrowding was defined as the index person sleeping with three or more additional persons
127 in the room. We obtained date of ART initiation and viral load at baseline from clinical records,
128 accepting viral loads completed up to two weeks post-enrolment. At endline we collected viral load
129 specifically for the study. We used the following validated questionnaires for mental health
130 assessment:
131 *The Shona Symptom Questionnaire (SSQ-14) [19, 21]*: The SSQ-14 is a measure of CMD developed
132 and validated in Zimbabwe which includes locally accepted idioms of distress. It consists of 14 binary
133 items and is scored from 0-14, with higher scores indicating more symptoms. A cutoff of ≥9 provides
134 88% sensitivity and 76% specificity among PLWH to diagnose CMD against a standardised
135 diagnosis of depression and/or general anxiety disorder [19], with good internal reliability (Cronbach

136	α=0.74).

137 *The Patient Health Questionnaire (PHQ-9) [19, 22]:* The PHQ-9 measures depression based on the
138 Diagnostic and Statistical Manual for Mental Disorders (DSM)-IV and DSM-V criteria. It consists of
139 nine items with 4-point Likert responses and scores from 0-27, with higher scores indicating worse
140 symptoms. The Shona language version was validated in primary care in Zimbabwe against a
141 diagnosis of depression. An optimal cut-off of ≥11 provided a sensitivity of 85% and specificity of
142 69%, with high internal reliability (Cronbach α=0.86) to detect depression.
143 *The Generalized Anxiety Disorder questionnaire (GAD-7) [19, 22]:* The GAD-7 is a screening tool for
144 general anxiety disorder which consists of seven items with four-point Likert responses and scores
145 from 0 to 21, with higher scores suggesting worse symptoms. The Shona language version was
146 validated in a Zimbabwean primary care context against a diagnosis of GAD. Its optimal cut-off of
147 ≥10 showed a sensitivity of 89% and specificity of 73% with high internal reliability (Cronbach
148 α=0.87). We defined the severity of anxiety at baseline according to the established GAD-7
149 categories (0-4 no, 5-9 mild, 10-14 moderate and 15-21 severe anxiety).
150 *The WHO Disability Assessment Schedule (WHO-DAS 2) [19]*: The WHO-DAS 2 is a 12-item score
151 for disability which ranges from 0-48, with higher scores indicating higher disability.

### 153 Friendship Bench Intervention

154 The FB intervention consists of six sessions of culturally adapted problem-solving therapy and simple
155 behavioural activation for depression delivered by a trained lay counsellor on a bench in a discreet
156 area outside the clinic [16, 17]. The first session was delivered on the day of enrolment with
157 subsequent weekly sessions. If a participant missed a session, the counsellor got in touch by phone
158 or SMS to encourage adherence to problem-solving therapy and re-schedule the next session.
159 Patients were also invited to a peer-led support group with an income generation component, known
160 as Circle Kubatana Tose. Twelve trained lay health counsellors employed by the City of Harare
161 Health Department delivered the FB intervention, supervised on a two-weekly basis by a mental
162 health care professional from the FB team. All counsellors were experienced in delivering the
163 intervention and had treated patients in the previous FB clinical trial [18] The initial training lasted
164 nine days and included knowledge on common mental disorders, counselling skills, problem-solving
165 therapy, and self-care. All lay counsellors attended a group discussion where lessons from the earlier
166 trial were shared, including how they addressed depression in PLWH. A five-day refresher training
167 on problem-solving therapy was then carried out with core modules on counselling PLWH and
168 management of participants with ‘red flags’ to empower the task-shifting approach and limit referral
169 to tertiary care.

### 171 Enhanced usual care

172 The comparison group received EUC consisting of nurse-led psychoeducation and assessment for
173 the need of further mental health care based on the Mental Health Gap Action Program (mhGAP) of
174 the WHO [23]. Further mental health care included the prescription of antidepressant medication by the
175 clinic nurse and strengthened referral to existing mental health care services for participants showing
176 a ‘red flag’ as defined above. These were also available in the FB group.

### 178 HIV care

179 All study participants received standard HIV care. In case of viral non-suppression nurse-led
180 enhanced adherence counselling was conducted in accordance with Ministry of Health
181 recommendations [24]. This consisted of three sessions on the benefits of ART and an in-depth
182 discussion on reasons for non-adherence with the option to refer to a physician.

### 184 Outcomes

185 The primary outcome was the proportion of participants with viral non-suppression at six months
186 follow-up, defined as a viral load ≥400 copies/mL or death. Secondary outcomes were mental health
187 symptoms assessed using the SSQ-14, PHQ-9, GAD-7, and WHO-DAS. These were analysed as
188 continuous scores and proportions screening positive as defined above (SSQ-14 ≥9, PHQ-9 ≥11,
189 GAD-7 ≥10 and WHO-DAS ≥20).

### 191 Statistical analysis

192 The study had 81% power to detect a difference in viral non-suppression at 6 months of 30% in the
193 EUC clinics vs 18% in the FB clinics, based on recruiting 750 participants from ten FB clinics and
194 250 from three EUC clinics, with an ICC of 0.01. The ZIMPHIA report 2016 [25] found that 15% of
195 adults in HIV care in Zimbabwe were non-suppressed; we estimated this would be twice as high
196 among people with CMDs.
198 Analyses were conducted at cluster-level due to the small number of clusters [26]. The primary
199 analysis was a cluster-level difference-in-difference approach assessed using linear regression of
200 cluster-level proportions or means on FB vs EUC, adjusted for baseline viral non-suppression (aDiD).
201 We used individual-level multivariable logistic regression to identify potential confounders (i.e.
202 baseline variables that were independently associated with treatment group and with viral non-
203 suppression at follow-up, adjusting for clinic using robust standard errors). We identified baseline
204 variables independently associated with loss-to-follow-up following the same statistical approach.
205 Potential confounders or variables associated with loss-to-follow-up were included in the initial aDiD
206 model if they changed the effect estimate by more than 10%. The p-value was estimated assuming
207 a t-distribution to account for the small degrees of freedom. The same approach was used for the
208 mental health outcomes, adjusting for the respective baseline mental health value. For the primary
209 outcome we also conducted an analysis stratified by baseline viral suppression.
211 We conducted two post-hoc sensitivity analyses of the primary outcome. First, we included only
212 participants with less than eight months follow-up to account for differences in follow-up time
213 between the treatment groups. Second, we excluded participants recruited in the last three months
214 of the recruitment period. During this time, data on reasons for non-eligibility were not collected,
215 which might have introduced a selection bias. All analyses were conducted in Stata version 17.0.

## 217 Results

### 218 Study flow

219 The original target was to enrol 75 participants per clinic in 10 FB clinics (N=750) and 83 per clinic
220 in three EUC clinics (N=249). However, fewer PLWH were attending per day than expected,
221 especially in four FB clinics and one EUC clinic. A key reason for this was relatives and friends of
222 PLWH coming to collect repeat prescriptions for them. Thus we reduced the sample size to six FB
223 and two EUC clinics, with enrolment targets of 80-100 per FB clinic (total N=500) and 100 per EUC
224 clinic (total N=200). Between August 2017 and July 2018, 2019 PLWH were screened in the six FB
225 clinics, of whom 543 (26.9%) were eligible, and 500 (92.1%) were enrolled (Fig 1). In the two EUC
226 clinics 817 PLWH were screened, of whom 212 (25.9%) were eligible, and 200 (94.3%) were
227 enrolled.
228 The main reason for non-eligibility in both FB and EUC clinics was not having CMD (n=1704). Even
229 though 99 participants reported hallucinations and 144 reported suicidal ideation when screened for
230 CMD at baseline, none of these patients were deemed to have suicidal intent or psychotic symptoms
231 by the study psychologist and thus we did not exclude any participants due to psychiatric reasons.
232 Data on reasons for exclusion was not available for participants screened in the last three months of
233 recruitment (May-July 2018), hence they are listed as ‘unknown’ (2.5% of those screened in the FB
234 clinics and 14.0% in the EUC clinics).

### 235 Fig 1: Participants living with HIV and CMD recruited into the study between August 2017 and

236 **July 2018**
238 Overall, 579 participants (82.7%) had viral load test results at both baseline and endline (81.4% and
239 86.0% in the FB and EUC group, respectively). The most common reason for non-availability of
240 baseline viral loads was collection outside the window period of two weeks post-enrolment. The
241 median duration from enrolment to follow-up viral load test was 6.5 months (IQR 5.3-7.8) in the FB
242 group and 8.1 months (IQR 6.8-11.7) in the EUC group (p<0.001). A total of 568 (81.1%) participants
243 had mental health outcome data at follow-up (81.2% and 81.0% in the FB and EUC groups,
244 respectively). The median duration of follow-up for the mental health outcomes was 6.5 months (IQR
245 5.2-7.2) in the FB group and 9.5 months (IQR 7.0-12.8) in the EUC group (p<0.001).

### 247 Study population characteristics

248 Table 1 shows baseline characteristics of the 579 participants with complete viral load data (i.e. at
249 baseline and endline) by treatment group. The mean age at enrolment was 40.6 years (SD 9.9
250 years). Most participants (82.0%) were female. About half the participants were married and most
251 had some secondary education (Table 1). Most (89.1%) participants stated that they had some
252 income being either employed, supported by the family and/or partner or running their own business
253 Most (89.6%) had at least one child and 37.3% were caring for other children than their own,
254 including grandchildren, with a similar distribution in both groups (data not shown). Of all participants,
255 13.6% lived in overcrowded conditions and 13.1% drank alcohol, but only five participants reported
256 they consumed alcohol daily. At baseline, 80 (13.8%) participants had viral non-suppression (14.3%
257 and 12.8% in the FB and EUC groups respectively). As shown in Table 1, median duration on ART
258 in both groups was similar, as was severity of CMD. Prevalence of major depression in the whole
259 sample (PHQ-9 ≥11) was 55.8% and of clinically relevant general anxiety disorder (GAD-7 ≥10) was
260 47.0%. Sixty-seven (11.6%) participants in the whole sample showed severe depression (PHQ-9
261 ≥20), and 132 (22.8%) severe generalised anxiety disorder (GAD-7 ≥15). Almost half of the
262 participants registered a ‘red flag’ (50.1% and 45.4% in the FB and EUC groups respectively). All
263 were assessed by senior counsellors but none were deemed in need of referral to tertiary care or
264 started on antidepressants during the study period.
266 Age group and time since ART initiation were associated with treatment group (Table 1) and endline
267 viral non-suppression (S1Table) and were deemed potential confounders, but did not act as
268 confounders as they changed the effect estimate by less than 10%. Viral load missingness was
269 associated with marital status, income, alcohol consumption, length of time on ART, and having an
270 SSQ red flag (S2Table). Of these, marital status, income and years on ART were also associated
271 with treatment group but did not act as confounders. Therefore, we did not adjust for any additional
272 baseline characteristics in the final analysis.

### 274 Table 1: Baseline characteristics of 579 participants with viral load testing at baseline and

275 **follow-up according to treatment group.**

|  |  | **EUC n (%) or mean (SD)**  N=172 | **FB n (%) or mean (SD)**  N=407 | **p** |
| --- | --- | --- | --- | --- |
| **SOCIODEMOGRAPHIC CHARACTERISTICS** | | | |  |
| **Gender** | Male | 45 (26.2%) | 59 (14.5%) | Χ^2^=11.2,  0.001 |
|  | Female | 127 (73.8%) | 348 (85.5%) |  |
| **Age group** | 18-29 | 19 (11.1%) | 53 (13.1%) | Χ^2^=3.2,  p=0.52 |
|  | 30-39 | 61 (35.5%) | 139 (34.2%) |  |
|  | 40-49 | 68 (39.5%) | 138 (33.9%) |  |
|  | 50-59 | 19 (11.1%) | 60 (14.7%) |  |
|  | 60-72 | 5 (2.9%) | 17 (4.2%) |  |
| **Marital status** | Married | 94 (54.7%) | 185 (45.5%) | Χ^2^=11.8,  p=0.003 |
|  | Single | 51 (29.7%) | 103 (25.3%) |  |
|  | Widowed | 27 (15.7%) | 119 (29.2%) |  |
| **Highest level of education achieved** | Incomplete primary | 8 (4.7%) | 23 (5.7%) | Χ^2^=9.4,  p=0.05 |
|  | Complete primary | 38 (22.1%) | 58 (14.3%) |  |
|  | Incomplete secondary | 57 (33.1%) | 119 (29.2%) |  |
|  | Complete secondary | 61 (35.5%) | 192 (47.2%) |  |
|  | Tertiary | 8 (4.7%) | 15 (3.7%) |  |
| **Income (N=698)** | No | 10 (5.8%) | 53 (13.1%) | Χ^2^=6.6,  p=0.01 |
|  | Yes | 162 (94.2%) | 352 (86.9%) |  |
| **Living in a house (N=574)** | No | 26 (15.4%) | 69 (17.0%) | Χ^2^=0.2,  p=0.63 |
|  | Yes | 143 (84.6%) | 336 (83.0%) |  |
| **Overcrowding^$^** | No | 148 (86.0%) | 352 (86.5%) | Χ^2^=0.02,  p=0.89 |
|  | Yes | 24 (14.0%) | 55 (13.5%) |  |
| **Drink alcohol** | No | 145 (84.3%) | 358 (88.0%) | Χ^2^=1.4,  p=0.23 |
|  | Yes | 27 (15.7%) | 49 (12.0%) |  |
|  | | | |  |
| **HIV RELATED CHARACTERISTICS** | | | |  |
| **Baseline HIV Viral load** | <400 copies/mL | 150 (87.2%) | 349 (85.8%) | Χ^2^=0.2,  p=0.64 |
|  | ≥400 copies/mL | 22 (12.8%) | 58 (14.3%) |  |
| **Years since ART initiation N=547** | 0 | 27 (15.7%) | 64 (15.9%) | Χ^2^=8.3,  p=0.14 |
|  | 1 | 23 (13.4%) | 46 (11.4%) |  |
|  | 2 | 10 (5.8%) | 51 (12.7%) |  |
|  | 3 | 20 (11.6%) | 30 (7.5%) |  |
|  | 4 | 13 (7.6%) | 33 (8.2%) |  |
|  | ≥ 5 | 79 (45.9%) | 178 (44.3%) |  |
|  | | | |  |
| **MENTAL HEALTH CHARACTERISTICS** | | | |  |
| **Depression severity (PHQ-9**  **score)** |  | 11.3 (5.1) | 12.1 (6.1) | t=-1.53,  p=0.13 |
| **Major depression (PHQ-9 ≥11)** | No | 77 (44.7%) | 179 (44.0%) | Χ^2^=0.03,  p=0.86 |
|  | Yes | 95 (55.2%) | 228 (56.0%) |  |
| **Anxiety severity (GAD-7 score)** |  | 9.2 (4.9) | 10.4 (5.4) | t= -2.58,  p=0.01 |
| **Anxiety disorder (GAD-7 ≥10)** | No | 97 (56.4%) | 210 (51.6%) | Χ^2^=1.1,  p=0.29 |
|  | Yes | 75 (43.6%) | 197 (48.4%) |  |
| **Risk identified *** | No red flag | 94 (54.7%) | 203 (49.9%) | Χ^2^=1.1,  p=0.29 |
|  | Red flag | 78 (45.4%) | 204 (50.1%) |  |
| **Disability (WHO-DAS ≥20)** | No | 135 (78.5%) | 317 (77.9%) | Χ^2^=0.02,  p=0.87 |
|  | Yes | 37 (21.5%) | 90 (22.1%) |  |
| EUC enhanced usual care  FB Friendship Bench | | | |  |

|  |  | **EUC n (%) or mean (SD)**  N=172 | **FB n (%) or mean (SD)**  N=407 | **p** |
| --- | --- | --- | --- | --- |
| PHQ-9 (Patient Health Questionnaire): 0 (no symptoms) to 27 (worst possible symptoms)  GAD-7 (Generalized Anxiety Disorder 7-item Scale): 0 (no symptoms) to 21 (worst possible symptoms)  WHO-DAS (World Health Organization Disability Assessment Schedule): 0 (no difficulty) to 48 (worst possible difficulty)  ^$^index person sleeps with three or more other persons in the room  * SSQ-14 score ≥11 and either suicidal ideation or hallucinations | | | |  |

### 277 Association of the Friendship Bench with viral non-suppression

278 The prevalence of viral non-suppression at baseline and endline stratified by clinic is shown in Fig
279 2. All FB clinics showed a reduction in viral non-suppression between baseline and endline, whereas
280 both EUC clinics showed an increase. Table 2 summarizes the proportion with viral non-suppression
281 at endline and the results of the aDiD analysis. Overall, 10.1% (41/407) of the participants in FB
282 group had viral non-suppression at endline as compared to 15.1% (26/172) at EUC group (aDiD= -
283 7.3%; 95%CI -14.7% to -0.01%; p=0.05). Among the 499 participants who were virally supressed at
284 baseline, prevalence of viral non-suppression at follow-up was lower in the FB group than the EUC

285	group. (2.9% vs 9.3%; DiD -6.5% (95% CI -10.5% to -2.4%), p=0.002). Among the 80 participants

286	with viral non-suppression at baseline, there was no evidence of a difference in viral non-suppression

287	at endline (53.5% vs 54.5%; p=0.93).

288

### 289 Fig 2: Proportion of patients with viral non-suppression at baseline and six months follow-

290 **up by clinic.**

### 292 Table 2: Viral non-suppression at follow-up by treatment group stratified according to

293 **baseline viral load. Results are difference-in-difference of proportions in viral non-**

### 294 suppression between groups from baseline to follow-up.

| **Baseline viral load** | **Treatment group** | **N** | **Follow-up viral load**  **≥400 copies/ml, n (%)** | **Difference-in-difference of proportion (95% CI)*** | **p-value** |
| --- | --- | --- | --- | --- | --- |
| NA | EUC | 172 | 26 (15.1%) | -7.3; (-14.7% to -0.01%) | 0.05 |
| NA | FB | 407 | 41 (10.1%) |  |  |
| <400  copies/ml | EUC | 150 | 14 (9.3%) | -6.5% (-10.5% to -2.4%) | 0.002 |
|  | FB | 349 | 10 (2.9%) |  |  |
| ≥400  copies/ml | EUC | 22 | 12 (54.5%) | -1.1% (-26.3% to 24.1%) | 0.93 |
|  | FB | 58 | 31 (53.5%) |  |  |

295

EUC: Enhanced usual care FB: Friendship Bench

*Difference-in-difference of proportions <0 means patients receiving the FB are less likely to have viral non-suppression at follow-up

**Association of the Friendship Bench with mental health**

297 Table 3 summarizes the associations of the FB with mental health. There was strong evidence that
298 participants in the FB group had lower prevalence of CMD at endline (SSQ-14 ≥9) than those in the
299 EUD group (aDID=-21.6%; 95%CI -36.5% to -6.7%; p=0.008). There was a similar pattern for
300 depression (PHQ-9 ≥11) and anxiety (GAD-7 ≥19), although the associations were not statistically
301 significant. There was also evidence of an association between FB and symptom severity of common
302 mental disorders (SSQ-14 score) at endline with (aDID= -1.90; 95%CI 3.50 to -0.31; p=0.02), but no
303 evidence of an association for the other mental health outcomes.

### 305 Table 3: Association of treatment group with mental health using cluster-level analysis

306 **adjusted for the respective baseline value. Results are adjusted DiD of proportions (binary**

### 307 outcomes) or in means (continuous outcomes) between treatment groups from baseline to

308 **follow-up.**

|  | **EUC**  **% (n/N)** | **FB**  **% (n/N)** | **Difference-in-difference of proportions (95% CI)*** | **p-value** |
| --- | --- | --- | --- | --- |
| Presence of common mental disorders (SSQ-14 cut-off ≥ 9) | 52.5% (85/162) | 27.6% (112/406) | -21.5% (-36.5% to -6.7%) | 0.008 |
| Presence of depression (PHQ-9 cut-off ≥ 11) | 31.5% (51/162) | 20.9% (85/406) | -0.81% (-28.7% to 12.6%) | 0.41 |
| Presence of anxiety (GAD-7 cut-off ≥ 10) | 28.4% (46/162) | 17.2% (70/406) | -7.7% (-31.0% to 15.6%) | 0.49 |
| Presence of disability (WHO-DAS cu-toff ≥ 20) | 14.8% (24/162) | 8.6% (35/406) | -2.9% (-17.7% to 11.9%) | 0.68 |
|  |  |  |  |  |
|  | **EUC**  **Mean (SD)** | **FB**  **Mean (SD)** | **Difference-in-difference in means (95% CI)^*^** | **p-value** |
| Severity of common mental disorders (SSQ-14 score) | 7.91 (4.17) | 5.55 (3.78) | -1.90 (-3.50 to -0.31) | 0.02 |
| Severity of depression (PHQ-9 score) | 8.20 (5.66) | 6.27 (5.44) | -2.07 (-5.24 to 1.10) | 0.18 |
| Severity of Anxiety (GAD-7 score) | 6.83 (4.83) | 5.31 (4.77) | -1.81 (-4.87 to 1.25) | 0.22 |
| Severity of disability (WHO-DAS score) | 10.85 (8.30) | 7.78 (7.61) | -1.36 (-6.52 to 3.80) | 0.58 |
|  |  |  |  |  |
| SSQ-14 (Shona Symptom Questionnaire): 0 (no symptoms) to 14 (worst symptoms) PHQ-9 (Patient Health Questionnaire): 0 (no symptoms) to 27 (worst possible symptoms)  GAD-7 (Generalized Anxiety Disorder 7-item Scale): 0 (no symptoms) to 21 (worst possible symptoms) | | | | |

WHO-DAS (World Health Organization Disability Assessment Schedule): 0 (no difficulty) to 48 (worst possible difficulty)

*Difference-in-difference of <0 means patients receiving the FB were less likely to have mental health problems at follow-up

### 310 Sensitivity analyses

311 The results for the primary outcome remained robust in both sensitivity analyses. When the sample
312 was restricted to those followed up within 8 months after enrolment (N=393) the aDiD was -8.1%
313 (95%CI -15.5% to -0.7%; p=0.04). Similarly, when it was restricted to those enrolled before May

314	2018 (N=497), the aDiD was -7.8% (95%CI -16.8% to 1.1%; p=0.08).

315

## 316 Discussion

### 317 Main findings

318 This comparative cohort study in 579 participants accessing ART through primary health care
319 showed that PLWH with co-morbid CMD who received the FB psychological intervention, were more
320 likely to maintain HIV viral suppression than those who received only standard of care mental health
321 treatment based on the WHO mhGAP [23]. Participants who were virally suppressed at baseline
322 were more likely to be virally suppressed at follow-up if they received FB compared to those who
323 received standard of care. However, there was no evidence of a difference in viral non-suppression
324 between mental health treatment groups in participants with viral non-suppression at baseline. This
325 suggests that FB helps PLWH maintain good adherence to ART and stay virally suppressed, but not
326 to newly achieve viral suppression. The burden of common mental disorders in this primary care
327 setting was high, with half the screened PLWH living with clinically relevant depression and/or
328 anxiety.

### 330 Findings of the study in larger research context

331 To our knowledge, this study is the largest comparative study conducted in sub-Saharan Africa to
332 assess the effect of a brief psychological intervention for CMD among PLWH who are either virally
333 suppressed or virally non-suppressed. The study was implemented during the scale-up of FB
334 through primary care in Zimbabwe, a low-income country with the fifth highest HIV prevalence
335 globally [27].
337 At the time we conducted this study, there was only preliminary evidence from African countries of
338 the potential to improve viral suppression through treating CMD, based on two pilot studies. One of
339 these conducted in Zimbabwe [28] piloted stepped care for depression integrated with enhanced
340 adherence counselling and one in Cameroon [29] used antidepressants. Both studies found that
341 treatment of depression was associated with increased prevalence of viral suppression, although
342 neither was powered to determine effectiveness [28, 29]. A pre-post study from Malawi compared
343 the FB for mild depression (and antidepressants for moderate to severe depression) with usual care
344 in 501 PLWH newly initiating ART with symptoms of depression (PHQ-9 ≥5) [30]. No evidence was
345 found that treatment of depression improved viral suppression as compared to usual care. A possible
346 explanation for this lack of effect may be that interventions for depression were poorly implemented,
347 there was no additional adherence counselling in the intervention arm and loss to follow-up was high.
348 A further reason could be that entry criteria for depression were too low. In the Malawi study only
349 25% of participants had a PHQ-9 score of 10 or above, compared to 63% in our study. Furthermore,
350 the study was restricted to those newly initiating ART, who may not yet be experiencing treatment
351 fatigue [31]. A recently published cluster-randomised trial from Uganda found that eight sessions of
352 group support psychotherapy, compared to group HIV education, improved depression and self-
353 reported ART adherence at 24 months in PLWH with major depression [32]. This is an encouraging
354 finding but it should be noted that the trial’s adherence measure was based on only one self-report
355 question, baseline adherence to ART was very high, and outcome assessors were unblinded, which
356 limits confidence in the adherence outcome. Viral suppression was greater (96%) in the intervention
357 arm, than the control arm (88%) but analysis was not stratified by baseline viral suppression.
359 Our findings that PLWH with viral non-suppression and CMD did not achieve viral suppression from
360 CMD treatment alone are in keeping with the evidence mainly from the United States that only
361 interventions with a specific adherence-related component resulted in improved adherence [33]. This
362 has been demonstrated recently in South Africa, where nurse-delivered cognitive behavioural
363 therapy for depression and adherence was effective in improving viral suppression as well as clinical
364 depression for virally unsuppressed PLWH [34]. PLWH who have depression and poor adherence
365 may need adherence-specific interventions to change their behaviour and re-attain viral suppression.

### 367 Strengths and limitations

368 We assessed CMD using three psychometric tests that were validated in the study setting [19, 21].
369 The PHQ-9 and the GAD-7 are standard tests to measure depression and anxiety in primary care
370 populations. Trained lay counsellors delivered FB in six primary health care centres in Harare. The
371 effect of FB on CMD and depression is already well documented, and has led to considerable scale-
372 up of the programme in Zimbabwe and other countries. This makes it an ideal intervention to test for
373 its potential effect on HIV viral suppression in PLWH.
375 A major limitation of the present study is the non-randomised design and consequently the risk of
376 bias due to confounding. Participants at the FB clinics were older, more likely to be female, more
377 likely to be widowed, less likely to have an income, and had been on ART for a longer period. To
378 decrease this risk we evaluated a comprehensive set of baseline characteristics as potential
379 confounders. We only found age and duration since ART initiation to be independently associated
380 with both treatment group and viral non-suppression at endline. These variables did not change the
381 effect estimate of the intervention on the primary outcome by more than 10% suggesting little
382 confounding.
384 Another limitation is that enrolment was lower than the planned sample size. Time and resource
385 constraints led us to close the study after including 500 participants in 6 clinics instead of 750
386 participants in 10 clinics in the FB group, and 200 participants in 2 clinics instead of 250 in 3 clinics
387 in the EUC group. The smaller than planned sample size might be an explanation for the lack of
388 statistically significant association between treatment group and depression or anxiety. All
389 associations between treatment group and mental health were in the same direction, suggesting
390 fewer mental health symptoms in the FB group.
392 Viral non-suppression was less prevalent than anticipated which limited our power to detect a
393 difference between groups at follow-up. Baseline prevalence of viral non-suppression was 13.8%,
394 which is similar to the 14.5% non-suppression found in a population-based survey conducted in
395 Zimbabwe [25]. We had expected that a sample with CMD symptoms would have higher baseline
396 viral non-suppression but this was not the case.
398 Other limitations include the difference in follow-up time between treatment groups and the lack of
399 data on eligibility during the last three months of recruitment. These differences in follow-up duration
400 occurred because of logistic challenges to data collection. The median follow-up was two months
401 longer in the EUC group than the FB group, giving the EUC group a longer period at risk in which to
402 develop viral non-suppression. This might have led to an underestimation of the effect of treatment
403 on viral non-suppression. In the sensitivity analysis restricted to participants who were followed up
404 within 8 months after enrolment, we found a slight increase in the reduction of the proportion of
405 participants at FB clinics with viral non-suppression as compared to the EUC clinics (-7.3% in the
406 main analysis and -8.1% in the subgroup analysis). The original study design specified 6 months’
407 follow-up. However, only 7 (4.1%) EUC participants were followed up within 6 months, so a restriction
408 at 8 months was used to permit a comparison between groups controlling to some extent for follow-
409 up time.

### 411 Implications for research and public health

412 We found evidence that the FB helped PLWH to maintain viral suppression, and to have better
413 mental health. It is vitally important to improve integration of mental health care into primary care for
414 people living with HIV in countries with high HIV burden[35]. More research is needed to understand
415 how to improve viral suppression in PLWH with co-morbid CMD. For instance, using problem-solving
416 therapy to help address barriers to adherence to ART, as well as to tackle depression could improve
417 both mental health and viral suppression in PLWH who are virally unsuppressed. An RCT is currently
418 underway to test this question, in Zimbabwe [36]. This is of public health importance, as viral
419 suppression plays a crucial role in ending the HIV pandemic. Additionally, research is needed to
420 understand which types of CMD in PLWH are most likely to respond to the FB intervention.
421 Qualitative research should be conducted, to deepen the understanding of adherence behaviour in
422 those living with HIV who have CMD, and to explore understand the needs of counsellors in
423 supporting people with adherence difficulties[3].

# 425 Conclusion

426 People living with co-morbid HIV and common mental disorders can benefit from a low-cost mental
427 health intervention to improve mental health outcomes and maintain viral suppression,..

# 429 Acknowledgements

430 We would like to acknowledge the City of Harare clinics staff to support the study with patient
431 recruitment, giving access to clinical data records and supporting the collection of blood samples to
432 measure viral load. We also would like to thank the research assistants Nyaradzo Goba, Tichaona
433 Gumunyu, Thembile Gola, and Portia Chiuyu for their support in data collection and coordination of
434 follow-up visits of the patients.

# 436 References

437 1. Cohen MS, Chen YQ, McCauley M, Gamble T, Hosseinipour MC, Kumarasamy N, et al.
438 Prevention of HIV-1 infection with early antiretroviral therapy. N Engl J Med. 2011;365(6):493-

439	505. Epub 2011/07/20. doi: 10.1056/NEJMoa1105243. PubMed PMID: 21767103; PubMed

440	Central PMCID: PMCPMC3200068.

441	2. Gulick RM. Adherence to antiretroviral therapy: how much is enough? Clin Infect Dis.

442	2006;43(7):942-4. Epub 2006/08/31. doi: 10.1086/507549. PubMed PMID: 16941381.

443 3. Costa-Cordella S, Rossi A, Grasso-Cladera A, Duarte J, Cortes CP. Characteristics of
444 psychosocial interventions to improve ART adherence in people living with HIV: systematic
445 review. PLoS Global Public Health. 2022;2(10).
446 4. Uthman OA, Magidson JF, Safren SA, Nachega JB. Depression and adherence to antiretroviral
447 therapy in low-, middle- and high-income countries: a systematic review and meta-analysis.

448	Curr HIV/AIDS Rep. 2014;11(3):291-307. Epub 2014/07/21. doi: 10.1007/s11904-014-0220-1.

449	PubMed PMID: 25038748; PubMed Central PMCID: PMCPMC4359613.

450	5. Langebeek N, Gisolf EH, Reiss P, Vervoort SC, Hafsteinsdottir TB, Richter C, et al. Predictors

451	and correlates of adherence to combination antiretroviral therapy (ART) for chronic HIV

452	infection: a meta-analysis. BMC Med. 2014;12:142. Epub 2014/08/26. doi:

453	10.1186/PREACCEPT-1453408941291432. PubMed PMID: 25145556; PubMed Central

454	PMCID: PMCPMC4148019.

455	6. Haas AD, Kunzekwenyika C, Hossmann S, Manzero J, van Dijk J, Manhibi R, et al. Symptoms

456	of common mental disorders and adherence to antiretroviral therapy among adults living with

457	HIV in rural Zimbabwe: a cross-sectional study. BMJ Open. 2021;11(7):e049824. Epub

458	2021/07/09. doi: 10.1136/bmjopen-2021-049824. PubMed PMID: 34233999; PubMed Central

459	PMCID: PMCPMC8264908.

460	7. Sudfeld CR, Kaaya S, Gunaratna NS, Mugusi F, Fawzi WW, Aboud S, et al. Depression at

461	antiretroviral therapy initiation and clinical outcomes among a cohort of Tanzanian women

462	living with HIV. AIDS. 2017;31(2):263-71. Epub 2016/11/12. doi:

463	10.1097/QAD.0000000000001323. PubMed PMID: 27835614; PubMed Central PMCID:

464	PMCPMC5177498.

465	8. Todd JV, Cole SR, Pence BW, Lesko CR, Bacchetti P, Cohen MH, et al. Effects of

466	Antiretroviral Therapy and Depressive Symptoms on All-Cause Mortality Among HIV-Infected

467	Women. Am J Epidemiol. 2017;185(10):869-78. Epub 2017/04/22. doi: 10.1093/aje/kww192.

468	PubMed PMID: 28430844; PubMed Central PMCID: PMCPMC5430940.

469	9. Lofgren SM, Bond DJ, Nakasujja N, Boulware DR. Burden of Depression in Outpatient HIV-

470	Infected adults in Sub-Saharan Africa; Systematic Review and Meta-analysis. AIDS Behav.

471	2020;24(6):1752-64. Epub 2019/11/14. doi: 10.1007/s10461-019-02706-2. PubMed PMID:

472	31720956; PubMed Central PMCID: PMCPMC7478178.

473	10. Brandt C, Zvolensky MJ, Woods SP, Gonzalez A, Safren SA, O'Cleirigh CM. Anxiety symptoms

474	and disorders among adults living with HIV and AIDS: A critical review and integrative

475	synthesis of the empirical literature. Clin Psychol Rev. 2017;51:164-84. Epub 2016/12/13. doi:

476	10.1016/j.cpr.2016.11.005. PubMed PMID: 27939443; PubMed Central PMCID:

477	PMCPMC5195877.

478	11. Hoare J, Sevenoaks T, Mtukushe B, Williams T, Heany S, Phillips N. Global Systematic Review

479	of Common Mental Health Disorders in Adults Living with HIV. Curr HIV/AIDS Rep.

480	2021;18(6):569-80. Epub 2021/11/19. doi: 10.1007/s11904-021-00583-w. PubMed PMID:

481 34792706; PubMed Central PMCID: PMCPMC8600343.
482 12. UNAIDS. Integration of mental health and HIV interventions: key considerations. Geneva:
483 World Health Organisation, 2022.
484 13. Nakimuli-Mpungu E, Musisi S, Smith CM, Von Isenburg M, Akimana B, Shakarishvili A, et al.
485 Mental health interventions for persons living with HIV in low- and middle-income countries: a
486 systematic review. J Int AIDS Soc. 2021;24 Suppl 2:e25722. Epub 2021/06/25. doi:
487 10.1002/jia2.25722. PubMed PMID: 34164926; PubMed Central PMCID: PMCPMC8222847.
488 14. Remien RH, Patel V, Chibanda D, Abas MA. Integrating mental health into HIV prevention and
489 care: a call to action. Journal of the International AIDS Society. 2021;24:e25748.
490 15. Sin NL, DiMatteo MR. Depression treatment enhances adherence to antiretroviral therapy: a

491	meta-analysis. Ann Behav Med. 2014;47(3):259-69. Epub 2013/11/16. doi: 10.1007/s12160-

492	013-9559-6. PubMed PMID: 24234601; PubMed Central PMCID: PMCPMC4021003.

493	16. Chibanda D, Mesu P, Kajawu L, Cowan F, Araya R, Abas MA. Problem-solving therapy for

494	depression and common mental disorders in Zimbabwe: piloting a task-shifting primary mental

495	health care intervention in a population with a high prevalence of people living with HIV. BMC

496	Public Health. 2011;11:828. doi: 10.1186/1471-2458-11-828. PubMed PMID: 22029430;

497 PubMed Central PMCID: PMCPMC3210104.
498 17. Abas M, Bowers T, Manda E, Cooper S, Machando D, Verhey R, et al. 'Opening up the mind':
499 problem-solving therapy delivered by female lay health workers to improve access to evidence-
500 based care for depression and other common mental disorders through the Friendship Bench
501 Project in Zimbabwe. International Journal of Mental Health Systems. 2016;10:39. Epub

502	2016/05/14. doi: 10.1186/s13033-016-0071-9. PubMed PMID: 27175215; PubMed Central

503	PMCID: PMCPMC4865009.

504	18. Chibanda D, Weiss HA, Verhey R, Simms V, Munjoma R, Rusakaniko S, et al. Effect of a

505	Primary Care-Based Psychological Intervention on Symptoms of Common Mental Disorders in

506	Zimbabwe: A Randomized Clinical Trial. Jama. 2016;316(24):2618-26. Epub 2016/12/28. doi:

507	10.1001/jama.2016.19102. PubMed PMID: 28027368.

508	19. Chibanda D, Verhey R, Gibson LJ, Munetsi E, Machando D, Rusakaniko S, et al. Validation of

509	screening tools for depression and anxiety disorders in a primary care population with high HIV

510	prevalence in Zimbabwe. Journal of Affective Disorders. 2016;198:50-5. Epub 2016/03/25. doi:

511	10.1016/j.jad.2016.03.006. PubMed PMID: 27011359.

512	20. von Elm E, Altman DG, Egger M, Pocock SJ, Gotzsche PC, Vandenbroucke JP, et al. The

513	Strengthening the Reporting of Observational Studies in Epidemiology (STROBE) statement:

514	guidelines for reporting observational studies. J Clin Epidemiol. 2008;61(4):344-9. Epub

515	2008/03/04. doi: 10.1016/j.jclinepi.2007.11.008. PubMed PMID: 18313558.

516	21. Patel V, Simunyu E, Gwanzura F, Lewis G, Mann A. The Shona Symptom Questionnaire: the

517	development of an indigenous measure of common mental disorders in Harare. Acta Psychiatr

518	Scand. 1997;95(6):469-75. Epub 1997/06/01. doi: 10.1111/j.1600-0447.1997.tb10134.x.

519 PubMed PMID: 9242841.
520 22. Spitzer RL, Kroenke K, Williams JB, Lowe B. A brief measure for assessing generalized anxiety
521 disorder: the GAD-7. Archives of Internal Medicine. 2006;166(10):1092-7.
522 23. World Health Organisation. mhGAP Intervention Guide for mental, neurological and substance
523 use disorders in non-specialized health settings. 2016.
524 24. Ministry of Health and Child Care. Guidelines for antiretroviral therapy for the prevention and
525 treatment of HIV in Zimbabwe. Harare: 2016.
526 25. Ministry of Health and Child Care. Zimbabwe Population-Based HIV Impact Assessment
527 (ZIMPHIA) 2015-2016: Final Report. 2019.
528 26. Hayes RJ, Moulton L. Cluster Randomised Trials. Boca Raton: Taylor & Francis; 2009.
529 27. UNAIDS. UNAIDS data 2019. 2019.
530 28. Abas M, Nyamayaro P, Bere T, Saruchera E, Mothobi N, Simms V, et al. Feasibility and
531 Acceptability of a Task-Shifted Intervention to Enhance Adherence to HIV Medication and
532 Improve Depression in People Living with HIV in Zimbabwe, a Low Income Country in Sub-

533	Saharan Africa. AIDS Behav. 2018;22(1):86-101. Epub 2017/01/08. doi: 10.1007/s10461-016-

534 1659-4. PubMed PMID: 28063075; PubMed Central PMCID: PMCPMC5758696.
535 29. Gaynes BN, Pence BW, Atashili J, O'Donnell JK, Njamnshi AK, Tabenyang ME, et al. Changes
536 in HIV Outcomes Following Depression Care in a Resource-Limited Setting: Results from a
537 Pilot Study in Bamenda, Cameroon. PLoS One. 2015;10(10):e0140001. Epub 2015/10/16. doi:
538 10.1371/journal.pone.0140001. PubMed PMID: 26469186; PubMed Central PMCID:

539	PMCPMC4607444.

540	30. Stockton MA, Udedi M, Kulisewa K, Hosseinipour MC, Gaynes BN, Mphonda SM, et al. The

541	impact of an integrated depression and HIV treatment program on mental health and HIV care

542	outcomes among people newly initiating antiretroviral therapy in Malawi. PLoS One.

543	2020;15(5):e0231872. Epub 2020/05/07. doi: 10.1371/journal.pone.0231872. PubMed PMID:

544	32374724; PubMed Central PMCID: PMCPMC7202614.

545	31. Claborn KR, Meier E, Miller MB, Leffingwell TR. A systematic review of treatment fatigue

546	among HIV-infected patients prescribed antiretroviral therapy. Psychol Health Med.

547	2015;20(3):255-65. Epub 2014/08/12. doi: 10.1080/13548506.2014.945601. PubMed PMID:

548	25110152; PubMed Central PMCID: PMCPMC4315727.

549	32. Nakimuli-Mpungu E, Smith CM, Wamala K, Okello J, Birungi J, Etukoit M, et al. Long-Term

550	Effect of Group Support Psychotherapy on Depression and HIV Treatment Outcomes:

551	Secondary Analysis of a Cluster Randomized Trial in Uganda. Psychosom Med.

552	2022;84(8):914-23. Epub 2022/09/27. doi: 10.1097/PSY.0000000000001128. PubMed PMID:

553	36162067; PubMed Central PMCID: PMCPMC9553261.

554	33. Mendez NA, Mayo D, Safren SA. Interventions Addressing Depression and HIV-Related

555	Outcomes in People with HIV. Curr HIV/AIDS Rep. 2021;18(4):377-90. Epub 2021/05/21. doi:

556	10.1007/s11904-021-00559-w. PubMed PMID: 34014446; PubMed Central PMCID:

557	PMCPMC8136266.

558	34. Safren SA, O'Cleirigh C, Andersen LS, Magidson JF, Lee JS, Bainter SA, et al. Treating

559	depression and improving adherence in HIV care with task-shared cognitive behavioural

560	therapy in Khayelitsha, South Africa: a randomized controlled trial. J Int AIDS Soc.

561	2021;24(10):e25823. Epub 2021/10/29. doi: 10.1002/jia2.25823. PubMed PMID: 34708929;

562	PubMed Central PMCID: PMCPMC8552453.

563	35. Abas M, O'Cleirigh C. Global mental health and the ambition to end AIDS by 2030. Lancet

564	Psychiatry. 2018;5(11):867-9. Epub 2018/10/14. doi: 10.1016/S2215-0366(18)30385-7.

565 PubMed PMID: 30314854.
566 36. Abas M, Mangezi W, Nyamayaro P, Jopling R, Bere T, McKetchnie SM, et al. Task-sharing
567 with lay counsellors to deliver a stepped care intervention to improve depression, antiretroviral
568 therapy adherence, and viral suppression in people living with HIV: study protocol for the
569 TENDAI randomised controlled trial. BMJ Open. 2022;In Press.

# 571 Supporting Information Captions

572 **S1 Table:** Associations between baseline characteristics and treatment group (exposure) or viral
573 non-suppression at endline (outcome) presented as ORs with corresponding p-values from
574 individual-level logistic regression analyses.
575 **S2 Table:** Associations between baseline characteristics and completeness of viral load (VL) data
576 presented as ORs with corresponding p-values from individual-level logistic regression analyses.

Figure_1	[Click here to access/download;Figure;Figure1.pdf](https://www.editorialmanager.com/pgph/download.aspx?id=123707&guid=6a6ee183-8bd8-4933-8123-e850a1e2f21c&scheme=1)

Figure_2	[Click here to access/download;Figure;Figure 2.pdf](https://www.editorialmanager.com/pgph/download.aspx?id=123708&guid=63901b57-153b-4866-a40c-f302615d8699&scheme=1)
