# Supplementary material for: Effect of a brief psychological intervention for common mental disorders on HIV viral suppression: A non-randomised controlled study of the Friendship Bench in Zimbabwe
Source: PLOS Glob Public Health. 2024 Jan 18;4(1):e0001492. doi: 10.1371/journal.pgph.0001492 (PMC10796049; doi:10.1371/journal.pgph.0001492)
Supplement: Attachment — Submitted filename: rebuttal_August14th.docx [file pgph.0001492.s006.docx]

10/8/23

Dear Editors,

**Re: Effect of a brief psychological intervention for common mental disorders on HIV viral suppression: a non-randomised controlled study of the Friendship Bench in Zimbabwe**

Thank you for your requested revisions of our paper.

1. A new unmarked version has been uploaded as a .doc file . labeled 'Manuscript'.
2. A marked-up copy of the manuscript that highlights changes made to the original version. Is uploaded as a separate file labeled 'Revised Manuscript with Track Changes'.
3. We noticed that you used "data not shown" in the manuscript.

**Response** – we removed this statement

4. We have noticed that you have a list of Supporting Information legends in your manuscript. **Response** – this list was wrong and is removed
5. In the online submission form, you indicated that your data will be submitted to a repository upon acceptance. We strongly recommend all authors deposit their data before acceptance, as the process can be lengthy and hold up publication timelines.

**Response** : the entire data will be made freely accessible if the manuscript is accepted for publication.

Yours sincerely,

Dr Vicky Simms and Prof Melanie Abas (on behalf of all authors)
